# Supplementary figures and images for: Diet Quality Affects the Association between Census-Based Neighborhood Deprivation and All-Cause Mortality in Japanese Men and Women: The Japan Public Health Center-Based Prospective Study
Source: Nutrients. 2019 Sep 12;11(9):2194. doi: 10.3390/nu11092194 (PMC6770038; doi:10.3390/nu11092194)

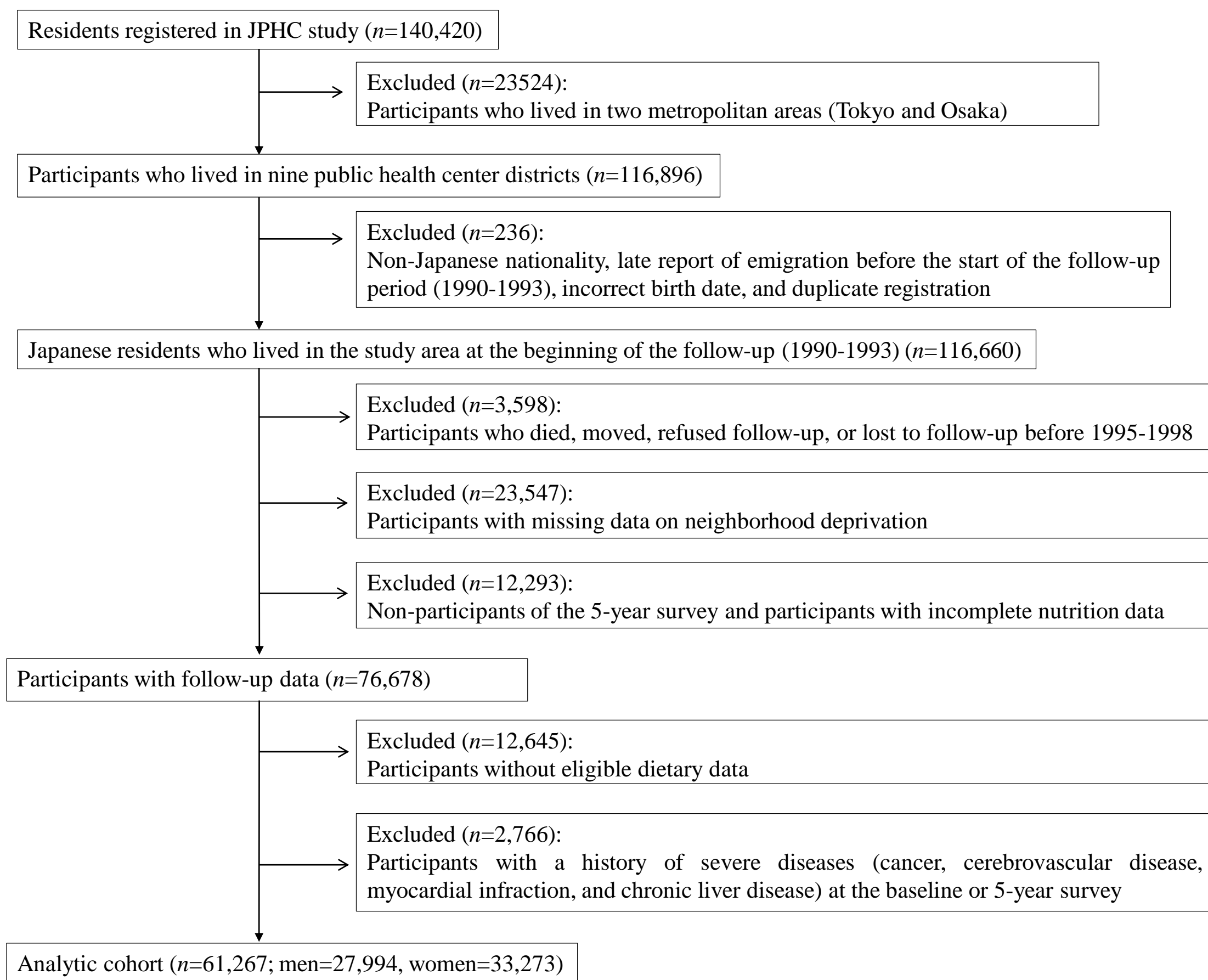

**Supplemental Figure 1.** Flowchart of the study population

Supplement: Supplementary file 1 [file nutrients-11-02194-s001.pdf]
